# Observations on Erysipelatous Fever, as It Occurred in Memphis, Tenn., in April and May, 1844, with a Report of 8 Cases

**Published:** 1845-01

**Authors:** Lewis Shanks

**Affiliations:** Memphis


Art. III.—*Observations on Erysipelatous Fever, as it occurred in Memphis, Tennessee, in April and May,* 1844, *with a report of eight cases.* By Lewis Shanks, M.D.

Preliminary to the observations we design making on the character and type of the erysipelatous fever, together with a report of the cases which occurred here, some general remarks on the character of diseases as modified by the seasons, by the clearing up and improvement of the country and the city, and the change in the habits of the people, might be interesting and useful, and necessary to a clear understanding of our views of the nature and treatment of this disease.

When the diversity of opinions both in Europe and America, of the nature and proper treatment of erysipelatous fever is considered, it must be attributed, either to the blinding influence of prejudice, or to the influence of season, climate, local atmospheric causes, or the difference in the constitutional tone and vigor of the subjects of the disease in different places and countries. If to both causes, and especially to the latter, this difference of opinion among highly distinguished members of the profession, may be attributed; an additional reason is afforded for tracing up the influence of the causes, that may have operated in determining the type of the disease, and indicating the proper treatment in the cases that occurred here. So full and complete are the general descriptions of this disease and its treatment furnished by the most able medical writers, &c., especially by Dr. Nunneley of Leeds, that little seems left to be done, except the investigation and explanation of local and epidemic influences, which modify the type and violence of the disease in different places and at different times, requiring a corresponding difference in the treatment.

Though we have proposed this extended consideration of the causes, which tended to make up the medical constitution of the atmosphere the present year, in this region of country, and to give character and modification in some degree, to all diseases, as being interesting and useful; we do not design more than a brief reference to these causes, and to their effects, as manifested in the general character of diseases.

We have not only been constantly impressed with the observation ourselves, but we have frequently heard the remark made by intelligent and observing physicians, that disease is more or less inflammatory—that there is more or less tendency to disease of particular organs—that depletion is more or less required—that purgatives are more useful, or tolerated worse, &c., some years than others, at the same period or season.

Inexplicable as may be the causes of the general constitution of the atmosphere of particular seasons producing these results, the correctness and truth of the remark will be admitted as unquestionable. While the diseases of Memphis and the surrounding country have each year exhibited some modification in their general character from this cause, a general but obvious change has been going on, probably since the first settlement of the country, and certainly for the last eight years. In the early settlement of the country and town, when but a small proportion of the lands was reduced to cultivation, and the timber was chiefly left standing upon the fields—when the town was like an encampment in the woods, closely surrounded by a heavy forest and thick undergrowth, the atmosphere was so loaded with humidity and miasm, that a state of atony and relaxation of the system was the universal condition of the permanent population who had been long exposed to it.

This condition of the system rendered it peculiarly liable to intermittent and remittent fevers, attended with congestion and great functional derangement of all the secretions, and in the fall and winter seasons especially, to violent and suddenly fatal attacks of congestion of the brain and lungs. Protracted cases of an inflammatory character of disease, were unknown except in recent settlers, arriving with the tone and elasticity of the system common in older, more elevated and improved countries, with a purer, dryer and more bracing atmosphere.

With the growth of the town and the improvement of the country—the clearing up and tramping the lands, rendering them firmer and dryer, the atmosphere has gradually been becoming less humid and filled with miasm, and more elastic and bracing; and the consequence is, that disease has gradually become less congestive and functional, and more inflammatory, complicated, and protracted in its character.

This change has been gradually and obviously manifesting itself every year, but in no year previously have the diseases of the winter, spring, summer, and autumn presented so decidedly an inflammatory character, and required so much depletion by venesection, and so rigid an antiphlogistic treatment as the present.

The statement of these general facts we have deemed proper, before the report of the cases of erysipelatous fever that occurred here, and the treatment that was instituted, because the depletion and antiphlogistic remedies mainly used and relied upon, were not only justified, but required by the medical constitution of the atmosphere of the season and this locality, modifying and rendering inflammatory a disease which in its general character is a *pyrexia* of excessive action, but deficient in the power and tone of the system.

Case I.—R—— W——, aged about 30, a carpenter, of steady habits and pretty good constitution, was attacked April 21st with slight hoarseness attended with pain in swallowing.

24th. Hoarseness and pain in deglutition very much increased; the epiglottis so enlarged and œdematous as to project up visibly. In deglutition there was not only great pain but part of the fluids passed into the trachea producing strangling and great distress. The pulse, tongue, fauces, skin, and general condition of the skin normal. No medicine had been taken but a purgative. Treatment—ordered a mercurial cathartic; rubefacient to the throat, and a stimulating gargle of infusion of red pepper, salt and vinegar.

25th. Fever developed; pulse about 90; œdema of the epiglottis increased; swallowing more painful and distressing; venesection ℥xxv.; tartarized antimony grs. viij. in solution to be taken through the night so as to produce repeated nausea, vomiting and purging; rubefacients and gargle continued.

26th.—7 A.M. Deglutition less painful and better. The tartar emetic has produced repeated vomiting and purging.

Remedies continued; the antimonial to be given in febrifuge doses.

5 P.M. Fever increased; venesection ℥xx.; cal. grs.xv.; stimulating foot-bath; gargle continued.

27th.—8 A.M. Deglutition much less painful and better. The œdematous condition of the epiglottis much relieved. Inflammation more diffused over the mouth, fauces and throat.

6 P.M. Considerable fever, but less swelling of the epiglottis; swallowing pretty good; ipecac. and cal. gr.ij. each to be given every two hours.

28th. The local disease of the epiglottis so relieved as not seriously to effect deglutition, which is still however attended with pain extending to all the muscles concerned in swallowing

The fever continued about a week longer, attended with pain in the pectoral and dorsal muscles, and occasionally paroxysms of spasmodic action in these muscles, so violent and painful as to require two more general bleedings, and repeated local bleedings with cups, followed by blisters, to relieve them. Auxiliary to the depletion, alteratives, diaphoretic anodynes, &c., were persevered in, until slight ptyalism was produced. The disease was gradually subdued and the patient recovered.

Of a very similar attack of this disease, we have been informed, Dr. Russell, of Huntsville, Ala., died some months since.

Case II.—Mr. R——, aged about 45, living in the same house, was attacked May 7th with phlegmonos erysipelas of the fore arm and arm. The limb swelled very much, attended with fever, furred tongue, and deranged biliary secretions. Suppuration occurred, extending between the muscles from near the wrist to the elbow. This case was treated chiefly by my partner, Dr. Frayser, (who assisted in the treatment of most of the cases), with mercurial purgatives and febrifuges, of either antimonials or ipecac. and cal. When suppuration took place, free incisions were made to discharge the matter, followed by bandaging and the cold dash. He recovered in three or four weeks.

Case III.—Mrs. R——, while dressing her husband's arm had a slight sore on her thumb, on the side of the nail. At that point inflammation commenced May 20th, and the thumb exhibited the appearance of a superficial whitlow.

23d. Thumb less swelled, but a red line extends from it along the course of the absorbents to the axilla. Complains of lassitude and aching in her limbs, with slight fever, and frequent but feeble pulse. Ordered cal. grs.xv.

24th.—7 A.M. Still walking about, but increase of fever and lassitude. Ordered febrifuge doses of cal. and ipecac., to be repeated every two hours.

25th.—6 P.M. Severe pain, tenderness and heat in the abdomen, (which had commenced the evening before), attended with rigors, dry tongue, thirst, frequent tense pulse, and cold extremities. Venesection ℥vj.; cal. grs.xv., morph. gr. $\frac{1}{3}$d, stimulating foot-bath, hot fomentations to the abdomen, to be applied frequently over a blistering plaster.

26th.—7 A.M. Pain less severe; pulse 130; extremities cold; tongue dry; very restless. ʒiss. oil, emollient poultice to the abdomen, and sinapisms to the extremities.

5 P.M. All tne symptoms worse. Great restlessness; prostration; internal heat; very frequent pulse, and extremities cold.

27th. Died in the night. Post-mortem examination objected to.

Cases IV. and V.—Were daughters of II. and III., aged about 12 and 14 years. They were attacked, one the 22d, the other the 24th of May. In both cases the attack commenced with slight chilliness, sore throat, and a remittent form of fever, and about the third or fourth day of the disease, an erysipelatous eruption occurred in both cases, on the side of the face, extending to and around the ear, and down the neck. Treatment:—One was bled, and both were treated with ipecac. and cal. in broken doses, to produce vomiting and purging, followed by mercurial cathartics, worked off with oil, and also febrifuge doses of antimonials or ipecac. Nitrate of silver applied freely to the eruption, which arrested its progress. They both recovered.

These were the first cases, and they all occurred in the same house. Shortly before they got sick, the house was plastered, and they lived and lodged in it during the time, moving from one room to another while the plastering was going on.

Case VI.—E—— R——, a girl, 14 years old, a visitor of case I., who had been removed near her place of residence, to his brothers, was attacked with sore throat and the erysipelatous eruption very much as cases IV. and V. were. She died about the 18th day of her disease, evidently from effusion in the brain. Having seen her near the close of her disease only, in consultation, the previous treatment cannot be particularly given.

Case VII.—J—— W——, brother of case I., to whose house he had been removed, was attacked about the 10th of May. Having called on the 12th to see case I., who was then convalescent, I saw him, and as he was in the care of another physician, only made a slight examination of his case, which exhibited all the characteristic symptoms of the others—frequent pulse, sore throat, and the erysipelatous eruption on the cheek and neck. Further than seeing a blistering plaster over the eruption, the treatment was not ascertained. Two days afterwards he died of effusion in the brain, as his friends reported there had been delirium some thirty-six hours previous to his death.

Case VIII.—On the 27th of May I was confined with this disease. For near a week previously I had felt languor and lassitude, with increased sensibility to cold, attended with wandering, gnawing pains, a dull headache and a very unpleasant metallic taste.

27th.—8 P. M. Headache; slight continued chilliness; pulse 110, and soreness of the throat. Treatment:—Hot foot-bath, warm drinks, followed by a cathartic.

28th.—5 P.M. Though the perspiration the night before was copious, and the cathartic had operated well in the morning, the fever, sore throat, restlessness and care all increased. Treatment by Dr. Frayser:—Venesection ℥xx., warm diluted drinks, pepper gargles, and rubefacients to the throat.

29th.—5 P. M. Fever continues; exacerbations in the evening; tonsils not much swollen, but from the condition of all the muscles concerned in deglutition and the cellular structure of the anterior part of the neck, swallowing is extremely painful, and almost impracticable. Erysipelatous eruption commenced on one side of the face, extending to and around the ear. Venesection ℥xxv., cal. grs.xv., liniment to the throat, emollient poultice, hot foot-bath, pepper gargle, &c.

30th. Symptoms not much changed; pulse 110 to 120; deglutition almost imperceptible; remedies continued. Nitrate of silver rubbed over the eruption.

31st. No improvement in the symptoms; eruption has not increased. Venesection ℥xx., cal. and aloes in broken doses, other remedies continued.

October 1st.—7 A.M. No better. Remedies continued by Drs. Christian and Frayser.

8 P.M. Take gr.ss. tartar emetic every two hours until vomiting or a decided impression is produced.

2d.—7 A.M. Better. The tartarised antimony, after two doses were taken, produced great nausea and relaxation, resulting in gentle perspiration, which was kept up by Dover's powder and cal. taken every two hours. Deglutition less painful and difficult and every way more comfortable. For the first time in four days a cup of drink could be swallowed, still, however, with some difficulty, but much less pain. My recovery from this hour was gradual, but as rapid as could be expected. An unpleasant and distressing symptom which had existed during the attack; the suspension of healthy secretion in the fauces and throat, accompanied with an excessively pungent alkaline taste, like a mixture of calcined magnesia and lime, continued for several days. This drynes; and peculiar feeling in the throat was gradually relieved by acid drinks, for which there was considerable craving.

Tonics and nourishing diet restored perfect health in a few weeks.

Although the first of these cases originated without the possibility of contagion, as no cases of the disease had occurred previously, all the other cases occurred among persons who were a good deal with the sick, and no case occurred in this vicinity out of the circle of the visitors of those who were laboring under the disease. It is, however, proper to state also, that many who visited these cases were not attacked with the disease.

The circumstances all considered go very far in establishing the fact, that the disease was communicated by contagion, and that the limited extent to which it spread, not amounting even to an endemic, was owing to the want of the atmospheric condition susceptible of being so infected by the contagion from a few cases, as to become the exciting cause of the disease beyond contact with the sick; and thus spread it either as an endemic, or as an epidemic fever, as it has prevailed within two years past in the New England States, and in the southern parts of Indiana and Ohio, according to the accounts given of it by Drs. Hall and Dexter, and Dr Sutton, published in the Philadelphia American Journal of Medical Sciences; and also in the southern part of Mississippi, as described by Dr. Pickett in the New Orleans Medical Journal.

We have expressed the opinion that erysipelatous fever, in its general character, is a *pyrexia* of excessive action but deficient tone and power of the system, and if it were proper in this article to do so, the preponderating weight of medical authority, from ancient down to modern writers, could be adduced in its support; but not caring for any display of this kind, we shall only consider the characteristic distinction between *pyrexiæ* and *phlegmasiæ* as classes of disease, as shewn by the experiments of Andral and others on the blood, in various diseases and conditions of the system; and assuming this disease to be a *pyrexia*, consider its peculiar character, from the specific cause which produces it, and from the condition of the system, and especially the blood, which gives rise to it, or is attendant upon it.

In *pyrexia*, the experiments of Andral have shown, that the proportion of fibrin is never increased, but often greatly diminished, while in *phlegmasia* the proportion of fibrin is always increased, and continues to increase until the inflammation is relieved. These relative proportions of the fibrin as a constituent of the blood, varying in amount in different cases and in the different stages of disease, are invariably characteristic of the two classes of disease, while the other constituents of the blood—the red globules—albumen and the serum may exist in various proportions in either class, only modifying the cases without giving invariable characteristic distinction to them.

In its general character, erysipelatous fever is peculiarly distinguished from inflammatory fever by the absence of adhesive inflammation, uniting and consolidating the serous membranes, and bounding and effectually limiting the extension of suppuration in the cellular structure. To the peculiar condition of the system—the state of the fluids, and the deficiency in the proportion of fibrin in the blood, precluding the effusion of plastic lymph to be formed into organised structure, is this characteristic difference mainly to be attributed. Instead of the adhesive inflammation and healthy suppuration that occurs in the phlegmasiæ, in erysipelatous fever, in the parts most impressed with disease there is effusion from the serous membranes, of bloody serum, or sero-purulent fluid with flakes of curdy lymph floating in it, and in the cellular structure profuse effusion of serum and lymph, producing great and diffuse swelling, and instead of healthy suppuration, the effused fluid degenerates into a sanious, or sero-purulent fluid, floating flakes of lymph, and extending often in the cellular structure between the muscles, their entire length; dissecting and separating the skin and muscles of a limb between the joints, or running the course of other muscles their entire length.

Taking this as a description of the peculiar general character of erysipelatous fever; the grades of the disease are so various, that it is sometimes so modified by various causes and circumstances, as the temperament of the patient, the natural vigor of constitution, the effects of kind and amount of food and clothing, habits of life, season of the year, age, the medical constitution of the atmosphere, &c., as to approximate genuine phlegmasia in its symptoms and pathological effects upon the system.

Admitting, therefore, willingly, all that may be claimed as to the different grades of this disease, and contending for diversity, and even opposite courses of treatment as being indicated in its different grades, we nevertheless incline to the opinion, that it is a disease sui generis, peculiar in its character, and produced by a specific cause, when it spreads to any considerable extent; and when it occurs sporadically in individual cases, is produced by the development of a peculiar constitutional susceptibility to the disease from ordinary causes.

After considerable investigation and much reflection on the subject, the treatment instituted and carried out in the cases that occurred here, would not be materially changed, because it was indicated and guided by the general inflammatory character of all diseases at that time, by the symptoms, the condition of the system, from the constitution, the habits, &c., of the different cases, without especial regard either to the name of the disease or to the high authority that erysipelatous fever, with diffuse inflammation, is a disease of feeble power and tone of the system, but excessive action.

The modifying causes and circumstances to which we have alluded, so influenced the disease in these cases as to approximate them near to the true phlegmasiæ, and the prompt and decided reduction of excessive action seemed indispensably necessary to prevent effusion in the brain in some of them, and in the cellular structure of the neck and about the throat, as well as to protect the brain, in my own case.

The case of the phlebitic form of the disease locating upon the peritoneum, in its course and speedy fatal termination, corresponded with all the cases seen by Drs. Hall and Dexter, where the disease affected the peritoneum. In the other two fatal cases treated by other physicians, neither venesection nor other decided antiphlogistic and febrifuge remedies were resorted to in the early stage of the disease, and their fatal termination was evidently from effusion in the brain.

Whilst it may be claimed and conceded on the one hand, that the treatment of these cases was not only based on correct pathological views, and the most successful that could have been adopted, on the other it is readily admitted, that where the inflammatory character or approximation exists in a less degree, and where there are evidences of an enfeebled state of the solids, and an impoverished and unstimulating condition of the fluids, an opposite course of treatment, consisting of tonics, diaphoretics, antiseptics, &c., would best and most successfully prevent or arrest effusion and sero-purulent degeneration, and guard the patient against the fatal effects and results of the disease.

Memphis, October 25, 1844.

